# Cooked oatmeal consumption is associated with better diet quality, better nutrient intakes, and reduced risk for central adiposity and obesity in children 2–18 years: NHANES 2001–2010

**DOI:** 10.3402/fnr.v59.26673

**Published:** 2015-05-27

**Authors:** Carol E. O'Neil, Theresa A. Nicklas, Victor L. Fulgoni, Maureen A. DiRienzo

**Affiliations:** 1 School of Nutrition and Food Sciences, Louisiana State University Agricultural Center, Baton Rouge, LA, USA; 2 Children's Nutrition Research Center, Department of Pediatrics, Baylor College of Medicine, Houston, TX, USA; 3 Nutrition Impact, LLC, Battle Creek, MI, USA; 4 Quadrant Nutrition, LLC, Hendersonville, NC, USA

**Keywords:** children, oatmeal, cooked cereal, NHANES, nutrient intake, diet quality, obesity

## Abstract

**Background:**

None of the studies of whole grains that have looked either at diet or weight/adiposity measures have focused exclusively on oatmeal.

**Objective:**

The objective of this study was to assess the association between oatmeal consumption and nutrient intake, diet quality, and weight/adiposity of children aged 2–18.

**Design:**

A nationally representative sample of children aged 2–18 (*N*=14,690) participating in National Health and Nutrition Examination Survey 2001–2010 was used. Intake was determined from a single 24-h dietary recall. Diet quality was measured using the Healthy Eating Index-2010 (HEI-2010). Covariate-adjusted regression analyses, using appropriate sample weights, were used to determine differences between oatmeal consumers and non-consumers for demographics, nutrient intakes, diet quality, and weight/adiposity measures (*p*<0.01). Logistic regression was performed to calculate odds ratios for weight measures and obesity (*p*<0.05).

**Results:**

Compared to non-consumers, oatmeal consumers were more likely to be younger and less likely to be smokers. Consumers had higher intakes of dietary fiber, vitamin A, thiamin, riboflavin, calcium, phosphorus, magnesium, iron, copper, and potassium, and significantly lower intakes of total, monounsaturated and saturated fatty acids, cholesterol, and sodium. Oatmeal consumers had higher dietary quality scores attributable to higher intakes of whole grains and lower intakes of refined grains and empty calories. Children consuming oatmeal were at lower risk for having central adiposity and being obese.

**Conclusions:**

Consumption of oatmeal by children was associated with better nutrient intake, diet quality, and reduced risk for central adiposity and obesity and should be encouraged as part of an overall healthful diet.

Oats (*Avena sativa* L.) have been identified as a whole grain (1), which is defined as a cereal grain that is intact, ground, cracked, or flaked kernel with the endosperm, germ, and bran present in the same relative proportions as the intact grain (2, 3). Compared with other cereal fibers, oats are rich in dietary fiber, which includes cellulose, arabinoxylans, and soluble fibers, especially β-glucan; oats also have relatively high levels of protein and unsaturated fats (4–6). β-Glucans are thought to be primarily responsible for the cholesterol-lowering property of oats (7), as well as improving appetite control and increasing satiety (8, 9). Those studies were done in adults and the effect on children has not been studied. Antioxidative components found in oats include vitamin E (tocopherols and tocotrienols), phenolic compounds, phytic acids, sterols, and flavonoids (10). Although some of the antioxidants in oats are heat labile, most are heat stable; this is an advantage since commercial oat products are often heat treated to inactivate enzymes (11) and are served hot as a cooked cereal.

For human consumption, oats are commonly processed into rolled oats by steaming and rolling pinhead oatmeal, which is made by cutting the whole kernel in half and sifting out any floury meal remaining (12). Oatmeal comes in several forms (i.e. steel cut, old fashioned, quick cooking, and instant), which are based on the level of processing and hence the cooking time. The nutrient composition of these different forms of oatmeal varies substantially. For example, one ounce equivalent (oz eq) (½ c) of cooked regular oatmeal provides 83 kilocalories (kcals), 0.32 g total sugars, 3 g protein, 2 g dietary fiber, 11 mg calcium, 1 mg iron, 32 mg magnesium, and 82 mg potassium; it is also virtually saturated fatty acid (SFA) and sodium free (13). Regular oatmeal is not micronutrient fortified (13). Instant oatmeal is fortified with B vitamins; 1 oz eq provides 158 kcals, 14.5 g sugar, 3.4 g dietary fiber, 4.3 g iron, 35 mg magnesium, and 211 mg sodium (13).

Consumption of whole grains by children in the Unites States is low (14, 15); O'Neil et al. (14) showed that the mean number of servings of whole grain consumed was 0.45, 0.59, and 0.63 for children/adolescents at the age of 2–5, 6–12, and 13–18 , respectively (14), which does not meet the current dietary recommendations (16, 17) of at least half the number of recommended grain servings be whole grain (17). The whole grain recommendation for children and adolescents varies; for children as young as 2 years, the recommendation is only 1.5 servings (oz eq), whereas the recommendation for children of age 9 and older is three servings per day (16, 17). Whole grain consumption by children has also been associated with increased nutrient intake, especially dietary fiber (14, 18, 19) and diet quality (14).

Overall, the consumption of whole grains has been associated with a modest reduced risk of cardiovascular disease, type 2 diabetes, and obesity (20). Most studies have been conducted in adults and little information is available for children; however, the American Academy of Pediatrics recommends fiber-rich diets, including those with whole grains to reduce the risk of cardiovascular disease (21) and to treat overweight or obesity in children (22). Whole grain consumption has also been associated with lower body mass index (BMI) -z scores (23) and lower weight/adiposity measures in children (24), but more studies are needed to confirm these limited findings.

None of the studies of whole grains that have looked either at diet or weight/adiposity measures have focused exclusively on oatmeal. Thus, the objective of this study was to assess the association between oatmeal consumption and nutrient intake, diet quality, and weight/adiposity of children aged 2–18.

## Methods

### Overview of the National Health and Nutrition Examination Survey

The National Health and Nutrition Examination Survey (NHANES) is an ongoing surveillance initiative conducted by the National Center for Health Statistics of the Centers for Disease Control and Prevention. The NHANES collects information about the diet and health of the non-institutionalized civilian population in the United States using a cross-sectional, nationally representative sample. NHANES survey data are collected via an in-home interview for demographic and basic health information, and a comprehensive diet and health examination was conducted in a mobile examination center. Detailed descriptions of the sample design, interview procedures, and physical examinations conducted are available online (25).

### Study participants

For these analyses, data from children aged 2–18 (*N*=14,690) participating in the NHANES 2001–2010 were combined to increase sample size (25). Survey response rates varied by data collection cycle, age, and gender and are available online (26). Federal law ensures confidentiality and protects individual NHANES participants from identification (27); thus, institutional review was not required (28).

### Demographics and dietary information

Demographic information, including age, gender, race/ethnicity, poverty index ratio, and current smoker status, was determined from the NHANES interview (29). Physical activity was determined from another interview (30). Alcohol consumption, along with other dietary information, was obtained from What We Eat in America, which used in-person 24-h dietary recall interviews administered using an automated multiple-pass method (31, 32). In 2001–2002, a single 24-h dietary recall was collected; however, beginning in 2003–2004, 2 days of intake were collected – the second by telephone. For consistency, only the data from the Day 1 (interviewer administered) dietary recall were used in this study. Proxies provided the 24-h dietary recall for children aged 2–5 and assisted children aged 6–11; older children provided their own recalls. Detailed descriptions of the dietary interview methods have been described previously (33).

To identify oatmeal consumers, the 24 food codes from United States Department of Agriculture's Food and Nutrient Database for Dietary Studies (FNDDS) relating to oatmeal as a cooked cereal (i.e. quick, instant, and regular oatmeal) were used. Individuals were classified as consumers if any oatmeal, as a cooked cereal, was ingested the day of the 24-h dietary recall. Energy and nutrient intakes were calculated using the FNDDS (34) versions 1.0, 2.0, 3.0, 4.1, and 5.0 for respective NHANES data sets 2001–2002, 2003–2004, 2005–2006, 2007–2008, and 2009–2010. The Vitamin D Addendum to United States Department of Agriculture (USDA) FNDDS 3.0 (35) was used to determine vitamin D intake. Intake from dietary supplements was not considered.

### Healthy Eating Index-2010

The USDA Healthy Eating Index-2010 (HEI-2010) was used to determine diet quality (36) of oatmeal consumers compared to those of non-consumers in terms of meeting recommended intakes of nine food groups and in meeting recommendations for food and dietary components to be limited in the diet, such as refined grains, sodium, and empty calories. Food group standards (37) and the development and evaluation of the HEI-2010 have been described previously (38, 39). The SAS code used to calculate HEI-2010 scores was downloaded from the Center for Nutrition Policy and Promotion website (40).

### Anthropometric measures

Height, weight, and waist circumference (WC), an index of central adiposity, were measured according to NHANES protocols (41). BMI was calculated as body weight (in kg) divided by height (in meters) squared (42). The BMI z-scores were calculated using the Statistical Analysis Software program for Centers for Disease Control and Prevention's Growth Charts (43). A BMI percentile between ≥85th and <95th was considered overweight, and ≥95th was considered obese. An elevated WC was defined as ≥ 85th percentile (43).

### Statistical analyses

Sampling weights and the primary sampling units and strata information (25) were included in all analyses using SAS v9.2 (SAS Institute, Inc., Cary, NC) and SUDAAN v 11 (Research Triangle Institute, Raleigh, NC). Least-squares mean and the standard errors were calculated using PROC REGRESS of SUDAAN. Covariate-adjusted regression analyses were used to determine differences between oatmeal consumers and non-consumers for nutrient intake, diet quality, and weight/adiposity measures. Statistical significance was *p*<0.01. Logistic regression analysis was used to determine differences in odds ratios (OR) for overweight, obesity, and WC between oatmeal consumers and non-consumers. Statistical significance was *p*<0.05. For all linear and logistic regressions, covariates were gender, race/ethnicity, age, poverty index ratio, physical activity levels, and current smoker and alcohol consumer status. Total energy intake (kcals) was also used as a covariate for nutrients.

## Results

Of the 14,690 participants, 418 (2.9%) consumed oatmeal on the day of the recall; among consumers mean oatmeal consumption was 228±8 g (0.96 cup equivalents; Table 1). Oatmeal consumers were significantly younger than non-consumers; and there was a lower prevalence of current smokers. The majority of oatmeal was consumed at breakfast (86.5±2.6%, data not shown).

**Table 1:** Demographic characteristics associated with oatmeal consumption in children aged 2–18: NHANES 2001–2010

Variable^a^	Consumers (LSM±SE)	Non-consumers (LSM±SE)	*p*
% Female	49.3±3.4	49.2±0.7	0.9770
Ethnicity			
% Non-Hispanic White	58.7±3.3	61.8±1.7	0.4066
% Non-Hispanic Black	18.8±2.1	13.7±0.9	0.0285
% Mexican-American	9.4±1.3	12.6±0.9	0.0483
Physical activity			
% Sedentary	15.0±2.6	13.1±0.5	0.4655
% Moderate	20.6±3.2	19.9±0.6	0.8408
% Vigorous	64.4±3.9	67.0±0.7	0.5134
% Current smokers	1.0±0.7**	6.4±0.4**	<0.0001
Age (years)	7.5±0.3**	9.8±0.1**	<0.0001
Poverty income ratio	2.6±0.1	2.5±0.0	0.6076
Alcohol (g)	0.3±0.2	0.5±0.1	0.3559
Oatmeal (g)	228±8	0±0	
*N*	418	14,272	
Weighted *N*	1,654,180	58,053,870	

a Differences assessed using z-scores.

** Significantly different. LSM, least-squares mean; SE, standard error.

As shown in Table 2, macronutrient intakes of oatmeal consumers varied significantly from those of non-consumers, but there were no differences in energy intake between the two groups. Oatmeal consumers consumed significantly less total fat, SFA, monounsaturated fatty acids, cholesterol, sodium, and more dietary fiber than non-consumers. Consumers also had significantly higher intakes of vitamin A, thiamin, riboflavin, calcium, phosphorus, magnesium, iron, copper, and potassium than non-consumers. Oatmeal consumers had a significantly higher HEI-2010 total score than non-consumers; specifically, higher components scores for whole grains, refined grains, and empty calories versus non-consumers (Table 3).

**Table 2:** Oatmeal consumption and energy, macronutrient, and micronutrient intakes in children aged 2–18: NHANES 2001–2010

Variable^a^	Consumers(LSM±SE)	Non-consumers (LSM±SE)	*p*
Energy (kcal)^b^	2,007±49	1,983±11	0.6432
Macronutrients			
Protein (g)	69.8±1.1	69.0±0.3	0.5229
Total fat (g)	67.5±1.1**	73.1±0.3**	<0.0001
Total SFA (g)	23.7±0.5**	25.8±0.1**	0.0005
Total MUFA (g)	24.3±0.5**	26.9±0.1**	<0.0001
Total PUFA (g)	13.8±0.4	14.3±0.1	0.2075
Cholesterol (mg)	188±8**	220±2**	0.0005
Dietary fiber (g)	16.2±0.5**	12.7±0.1**	<0.0001
Total sugars (g)	143±3	137±1	0.0567
Added sugars (tsp eq)	19.6±0.9	20.8±0.2	0.1543
Micronutrients			
Vitamin A (RAE mcg)	854±32**	577±7**	<0.0001
Vitamin D2+D3 (mcg)	5.7±0.3	6.0±0.1	0.4056
Thiamin (mg)	1.7±0.04**	1.5±0.01**	0.0004
Riboflavin (mg)	2.3±0.1**	2.1±0.02**	0.0070
Niacin (mg)	21.3±0.6	20.7±0.1	0.2781
Folate (DFE mcg)	541±19	531±5	0.5925
Vitamin B_12_ (mcg)	4.5±0.2	5.1±0.1	0.0173
Total choline (mg)	249±6	248±2	0.8774
Calcium (mg)	1,123±30**	1,007±8**	0.0003
Phosphorus (mg)	1,346±21**	1,247±6**	<0.0001
Magnesium (mg)	282±6**	226±1**	<0.0001
Iron (mg)	17.1±0.4**	14.3±0.1**	<0.0001
Zinc (mg)	10.6±0.3	10.6±0.1	0.8901
Copper (mg)	1.2±0.04**	1.0±0.01**	0.0002
Selenium (mcg)	96.0±1.8	91.4±0.5	0.0173
Sodium (mg)	3,006±46**	3,133±15**	0.0083
Potassium (mg)	2,411±50**	2,205±15**	0.0002

a All variables except energy adjusted for sex, race/ethnicity, age, poverty income ratio, physical activity level (sedentary, moderate or vigorous), current smoking status, alcohol consumption and energy intake

b Adjusted for sex, race/ethnicity, age, poverty income ratio, physical activity level (sedentary, moderate, or vigorous), current smoking status, and alcohol consumption.

** Significantly different. LSM, least-squares mean; SE, standard error; SFA, saturated fatty acids; MUFA, monounsaturated fatty acids; PUFA, polyunsaturated fatty acids; tsp eq, teaspoon equivalent; RAE, retinol activity equivalents; DFE, dietary folate equivalents.

**Table 3:** Oatmeal consumption and diet quality (Healthy Eating Index-2010) in children aged 2–18: NHANES 2001–2010

Variable^a^	Consumers (LSM±SE)	Non-consumers (LSM±SE)	*p*
HEI-2010 total score	54.0±1.1**	43.3±0.3	<0.0001
HEI components			
Total vegetables	2.1±0.11	2.1±0.03	0.9302
Greens and beans	0.6±0.10	0.6±0.02	0.8602
Total fruit	2.9±0.15	2.5±0.04	0.0155
Whole fruit	2.4±0.17	2.0±0.05	0.0512
Whole grains	6.9±0.19**	1.7±0.04**	<0.0001
Dairy	7.0±0.22	7.0±0.05	0.8294
Total protein foods	3.2±0.13	3.5±0.02	0.0670
Seafood/plant protein	1.5±0.16	1.3±0.03	0.5248
Fatty acid ratio	3.9±0.28	3.7±0.04	0.5101
Sodium	5.5±0.20	5.0±0.05	0.0186
Refined grains	6.9±0.19**	5.1±0.06**	<0.0001
Empty calories^b^	11.1±0.52**	8.9±0.11**	<0.0001

a Adjusted for sex, race/ethnicity, age, poverty income ratio, physical activity level (sedentary, moderate, or vigorous), current smoking status, and alcohol consumption

b calories from solid fats, alcohol, and added sugars; threshold for counting alcohol is >13 g/1,000 kcal.

** Significantly different (*p*<0.01). LSM, least-squares mean; SE, standard error.

Oatmeal consumers had significantly lower WC, but there were no significant differences in weight, BMI, BMI z-score, or BMI-for-age percentile (Table 4). Compared to non-consumers, oatmeal consumption was associated with a 40% lower risk of being obese and a 64% lower risk of having an elevated WC (Table 5).

**Table 4:** Oatmeal consumption and weight, waist circumference, and BMI measures in children aged 2–18: NHANES 2001–2010

Variable^a^	Consumers (LSM±SE)	Non-consumers (LSM±SE)	*p*
Weight (kg)	40.3±0.6	41.7±0.2	0.0174
Waist circumference (cm)	66.2±0.6**	67.9±0.2**	0.0032
BMI (kg/m^2^)	19.4±0.2	19.8±0.1	0.0497
BMI z-score	0.33±0.07	0.46±0.02	0.0453
BMI percentile	58.7±1.8	62.2±0.6	0.0368

a Adjusted for sex, race/ethnicity, age, poverty income ratio, physical activity level (sedentary, moderate, or vigorous), current smoking status, and alcohol consumption.

** Significantly different (*p*<0.01). LSM, least-squares mean; SE, standard error.

**Table 5:** Odds ratios for elevated waist circumference, overweight, and obesity in oatmeal-consuming children aged 2–18: NHANES 2001–2010

	Consumers^a^			
		
Variable^b^	OR	LCL	UCL	*p*
Overweight	0.83	0.57	1.21	0.3353
Obese	0.60*	0.38	0.94	0.0257
Overweight or obese	0.67*	0.47	0.9527	0.0262
Elevated WC	0.36*	0.14	0.95	0.0399

a Non-consumers was the comparison group

b adjusted for sex, race/ethnicity, age, poverty income ratio, physical activity (sedentary, moderate, or vigorous), current smoking status, and alcohol consumption.

* Odds ratio confidence interval does not include 1.00 (*p*<0.05). OR, odds ratio; LCL, lower 95% confidence limit; UCL, upper 95% confidence limit.

## Discussion

This was the first study to assess the associations between oatmeal consumption and nutrient intakes, diet quality, and weight/adiposity measures in children. Oatmeal consumption was associated with better nutrient intake and diet quality. Central adiposity was lower in oatmeal consumers, and they had a lower risk for being obese and having an elevated WC than non-consumers.

The number of oatmeal consumers in this study was low, with only 2.9% of children consuming cooked oatmeal on the day of the 24-h dietary recall; however, this figure represented more than a million and a half children. Daily mean oatmeal intake among consumers was relatively high and consisted of nearly 2 oz eq of whole grain. For children aged 2–8, this exceeded their daily recommended amount of whole grains, and for older children, 9–18 years, this met at least half of the recommendation for whole grains (16). Another NHANES study has shown that oatmeal constitutes 12% of whole grain consumption in children, with the major sources being ready-to-eat cereal (25%) and yeast bread/rolls (24%) (18).

Not surprisingly, this level of whole grain consumption was reflected in a higher HEI-2010 component score for whole grains and a higher intake of dietary fiber in oatmeal consumers compared to non-consumers. These findings confirm previous studies using NHANES data that showed that whole grain consumption was associated with a higher dietary fiber intake than that seen in non-consumers (14, 18). Regardless of oatmeal consumption or that of other foods high in dietary fiber, such as whole grain/fruit/vegetables, few children meet the recommendations for dietary fiber (44). Choosing oatmeal as a food option may be an effective strategy to help children improve their intake of both whole grains and fiber.

Health effects of dietary fiber have not been well studied in children (45, 46). Studies do suggest that increased fiber intake has been found to be associated with better diet quality and lower risk for overweight or obesity (17, 18, 24). Constipation is a serious health problem in children; however, using fiber as a treatment for constipation, study results have been conflicting (47–50). However, the North American Society for Pediatric Gastroenterology, Hepatology and Nutrition (51) and the American Association of Pediatrics (52) consider the current evidence too weak to support a recommendation for fiber supplementation in the treatment of constipation; however, they do support a balanced diet, including whole grains and other high fiber foods as part of the treatment. Additional studies of the health benefits of consuming high fiber foods, such as oatmeal, by children are needed.

Oatmeal consumers also had lower intakes of total fat, SFA, and cholesterol. These findings, coupled with the higher intake in dietary fiber, could lead to a reduced risk of coronary heart disease. Although oatmeal contains β-glucan, a soluble fiber that has been shown to lower cholesterol levels (53), studies in children are lacking.

The intakes of many micronutrients were significantly higher among oatmeal consumers than non-consumers. Regular cooked oatmeal is not micronutrient fortified; however, the instant varieties are vitamin fortified (13). A limitation of this study is that no information was available on the number of children who consumed instant versus other forms of oatmeal.

Cereals are usually consumed with milk (54) and the ‘package’ of cereal and milk can help improve the nutrient intake profile of consumers. A number of the nutrients consumed in higher amounts by oatmeal consumers could reflect milk consumption; these included vitamin A, riboflavin, calcium, phosphorus, and potassium (13). However, neither vitamin D nor dairy, as an HEI-2010 component, were higher in oatmeal consumers; thus, the source of the nutrients consumed in higher amounts by oatmeal consumers is unclear and may simply reflect an overall healthier eating pattern as shown by the higher HEI-2010 scores. It is important to note that of the four nutrients identified as being of public health concern (17), consumption of oatmeal was associated with greater consumption of three – dietary fiber, calcium, and potassium. Oatmeal consumers had a mean sodium intake that was lower by 127 mg/day compared to non-consumers; however, this was not enough of a difference to be reflected in a better sodium HEI-2010 component score for oatmeal consumers. The mean sodium intakes of both consumption groups exceeded the Dietary Reference Intake of 1,500 mg/day (55).

Diet quality, as reflected by HEI-2010 scores, was higher in oatmeal consumers than in non-consumers. The driving factors here were higher component scores for whole grains, refined grains, and empty calories in oatmeal consumers. It is important to note that refined grains and empty calories are ‘reverse scored’, so the higher the value the greater the contribution to higher overall diet quality (39). O'Neil et al. (14) also showed that consumption of whole grains was associated with better diet quality among children. In that study, the average consumption of whole grains was approximately 0.5 serving/day for children aged 2–5 and approximately 0.66 serving/day among children aged 6–18. When the population was stratified by the number of servings of whole grain foods consumed per day, intakes of fiber increased and intakes of total fat, SFA, and cholesterol decreased as whole grain servings increased. Diet quality (HEI-2005) also increased with the number of whole grain servings consumed per day. Clearly, strategies are needed to encourage greater consumption of whole grain foods among children, not only as a means to increase fiber intake but also as a way to increase the overall diet quality.

Mean energy intakes and levels of physical activity did not differ between oatmeal consumers and non-consumers on the day of the survey; however, oatmeal consumers had a lower mean WC, an indicator of central adiposity. Oatmeal consumers were also less likely to have elevated WC or to be obese. Although these findings suggest that the proportion of children classified as obese was greater in the non-consumers compared to the oatmeal consumers, the mean BMI or BMI z-score did not differ between the groups. Since there were no differences in energy intakes on the day of the 24-h dietary recall or in physical activity, the lower likelihood of obesity among oatmeal consumers merits further study. One possibility is that oatmeal consumers may have an overall healthier eating pattern and lifestyle, which over time would result in a lower risk for obesity; this possibility is supported by the higher fiber and whole grain intakes.

### Advantages and limitations

The strengths of this study were that it included a large sample size with a nationally representative sample of children. The NHANES has carefully controlled protocols and screens 24-h dietary recalls confirming that they are valid and complete; the NHANES also uses the multiple-pass method to obtain dietary intake, which is the best dietary assessment method available for large-scale epidemiologic studies. Twenty-four hours dietary recalls do have several intrinsic limitations. They may not represent usual intake; however, the mean of the intake distribution drawn from a large, representative sample of a group is not affected by day-to-day variation (56). Twenty-four hours dietary recalls are memory dependent, and under- and over-reporting may occur. In proxy-assisted recalls of children, parents may know what their children consume at home, but they may not know what their children consume outside the home, for example in school or day care (57, 58). There is also the possibility with a single 24-h dietary recall that the children were misclassified as oatmeal consumers. Finally, cause-and-effect relationships cannot be determined from cross-sectional epidemiologic data.

## Conclusion

This study shows that consumption of oatmeal by children aged 2–18 was associated with better nutrient intake and diet quality as shown by higher HEI-2010 scores, primarily driven by greater consumption of whole grains and lower intakes of refined grains and empty calories. Oatmeal consumers also were at lower risk for central adiposity or being obese. Healthcare professionals should consider recommending that children incorporate oatmeal as part of an overall eating plan to improve diet quality and reduce risk for obesity.
